# Trends in nephrology: from nihilism to targeted treatment of antibody-mediated rejection

**DOI:** 10.1093/ndt/gfag011

**Published:** 2026-01-24

**Authors:** Josef Filip Pickl, Rainer Oberbauer

**Affiliations:** Department of Internal Medicine, Division of Nephrology, Medical University of Vienna, Vienna, Austria; Department of Internal Medicine, Division of Nephrology, Medical University of Vienna, Vienna, Austria

**Keywords:** AMR, CD38, CD40L, kidney, transplantation

## Abstract

Antibody-mediated rejection (AMR) is among the leading causes of kidney transplant attrition. Traditional AMR therapy was based on interventional studies that were small and often uncontrolled. Although a blockade of the interleukin (IL)-6 pathway failed to prevent allograft loss in a phase 3 trial, anecdotal evidence had previously suggested potential benefit. Likewise, anti-CD20 antibodies have not been shown to improve AMR outcomes in terms of graft survival. Imlifidase, which cleaves all immunoglobulin Gs, and hence also all donor-specific antibodies, showed a higher rate of graft loss in the experimental group of an AMR study. Terminal complement inhibitors showed inconsistent efficacy in underpowered trials; currently, C3 inhibitors are a promising focus for complement-targeted strategies against AMR. Anti-CD38 antibodies currently represent the most promising novel therapy option for AMR based on encouraging phase 2 study results. We furthermore discuss genetically engineered CD38 knock-out multitarget natural killer cells, expressing anti-B cell maturation antigen (BCMA) CAR, IL-15RF and hnCD16, administered in combination with daratumumab (anti-CD38) in this context. This approach represents a cheaper and safer alternative to chimeric HLA antigen receptor (CHAR) T cell therapy. CHAR T cells showed a high specificity in recognizing their respective target anti-HLA class I B cell receptors *in vitro*. Quantitative results from *in vivo* trials are pending. Likewise, we hypothesize the use of bi-specific T cell engagers such as CD19 blinatumomab or BCMA teclistamab for the treatment of AMR. In addition, clinical studies investigating conversion from calcineurin inhibitors to co-stimulation therapies such as anti-CD40L antibodies may reduce dnDSAs and AMR. In summary, anti-CD38 antibodies currently constitute the drug class with the strongest evidence for AMR treatment. To date, most CD38 antibodies have been shown to be safe, even after several years of administration in patients with multiple myeloma.

## THE TRADITIONAL THERAPY

Antibody mediated rejection (AMR), as broadly defined in this editorial to include active and chronic active AMR, is one of the leading causes of kidney transplant rejection [1]. As reflected in recent versions of the Banff classification, a diverse set of pathomechanisms can give rise to AMR phenotypes, however this editorial is primarily focusing on antibody-mediated phenotypes [2]. Current treatment regimens for AMR include plasmapheresis combined with intravenous immunoglobulins (IVIG) for temporary elimination of donor-specific antibodies (DSAs). Some transplant centres add rituximab (anti-CD20) or bortezomib (proteasome inhibitor) to their regimen [3]. There is weak evidence to support the benefit of plasmapheresis although a recent paper of a small randomized controlled trial (RCT) from Australia reported that 1 g/kg/month of IVIG therapy was associated with stabilization in allograft histology and estimated glomerular filtration rate (eGFR) after 12 months in 15 kidney transplant recipients with chronic active AMR [4]. Most studies have not been controlled and did not assess the frequency and volume of plasma exchanged. Furthermore, rituximab and bortezomib have both failed to achieve positive outcomes, such as a reduced GFR slope or longer graft survival, when added to plasmapheresis/IVIG as AMR treatment [5, 6]. One current trial is comparing the efficacy of plasma exchange with double plasmapheresis in AMR (NCT03436134). Historically, an abundance of mostly small, interventional studies, often lacking control groups, has polluted the field of AMR research rather than delivering clear evidence and suggesting standardized treatment protocols. This inconsistency of evidence acted as a hurdle for progress, until now.

## NOVEL DATA FITTING THE TRADITIONAL THERAPY

Recently, alternative immunological targets have become the focus of trials, however pursuing similar goals as traditional approaches such as anti-interleukin (IL)-6 and anti-IL-6 receptor blockade, reduction of immunoglobulin G (IgG) and plasma cell depletion (Fig. 1, Table 1). The anti-IL-6 antibody clazakizumab showed promising results in small phase 2 trials (NCT03380377, NCT03444103) considering both, safety and efficacy, yet a larger phase 3 RCT (NCT03744910) with 194 randomized patients was terminated at the 1-year interim analysis due to what the authors described as ‘unlikeliness to meet the primary outcome’, which initially was all-cause graft failure and has been changed to eGFR slope as Food and Drug Administration accepted surrogate parameter of allograft loss.

**Figure 1:**
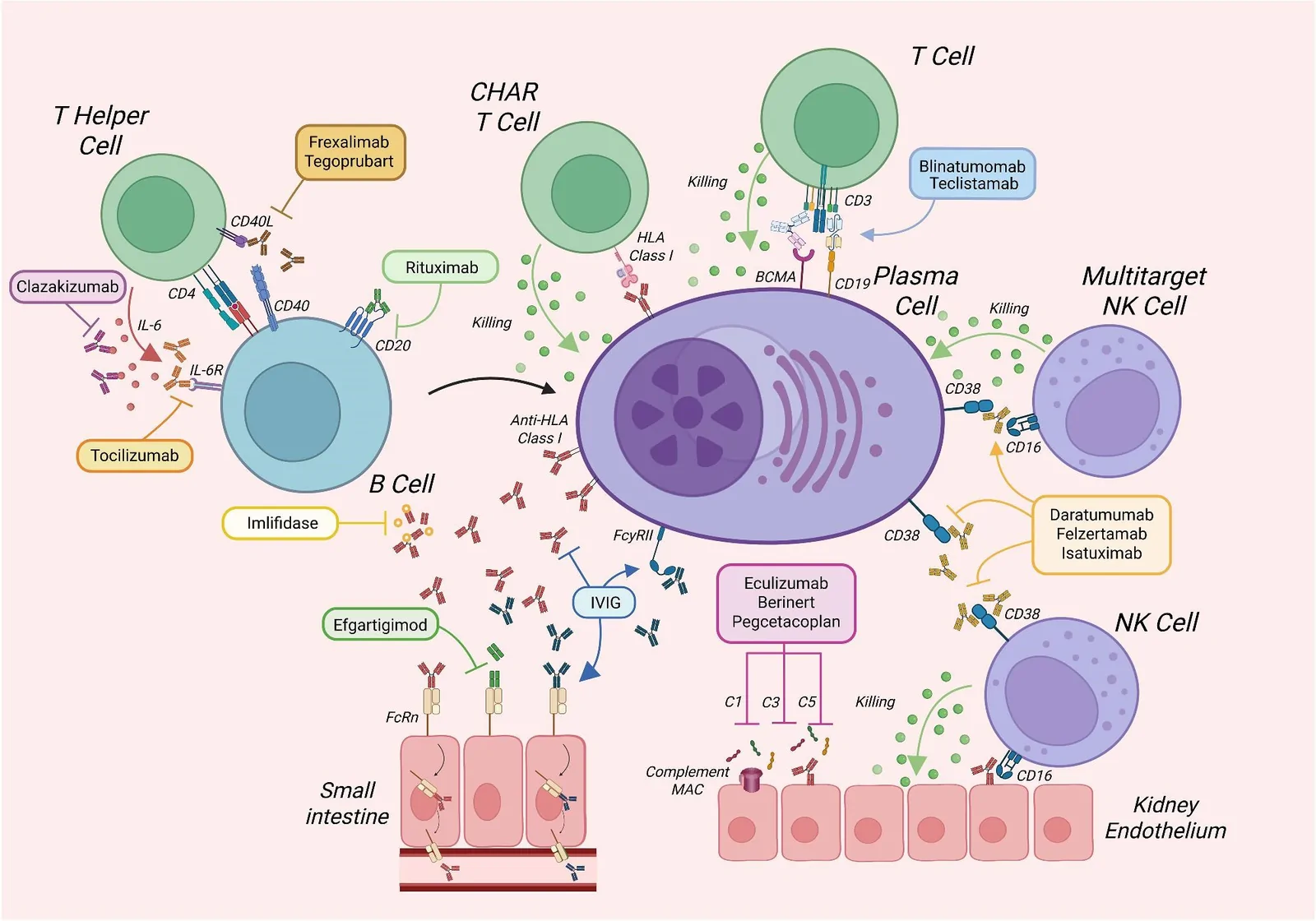
Pathophysiology of AMR and respective therapy targets. Counterclockwise: efgartigimod inhibiting the neonatal Fc receptor (green); IVIG binding neonatal Fc receptor and Fc-gamma receptor II (blue); berinert, pegcetacoplan and eculizumab inhibiting C1, C3 and C5, respectively (pink); daratumumab, felzertamab and isatuximab targeting CD38 (yellow); multitarget NK cell recognizing Fc of daratumumab bound to target cell’s CD38; blinatumomab binding CD19 and CD3-epsilon and teclistamab binding BCMA and CD3-epsilon (light blue); CHAR binding anti-HLA class I B cell receptor; rituximab targeting CD20 (light green); frexalimab and tegoprubart inhibiting CD40L (brown); clazakizumab neutralizing IL-6 (IL6) (violet); tocilizumab blocking IL-6R; imlifidase cleaving IgG (alloantibodies); anti-HLA class I antibodies (red). Created in Biorender by *Josef Pickl*.

**Table 1:** Ongoing clinical trials and studies of interest.

Treatment and trial name	Target	Study type	Participants	Trial number	PMID/DOI
The traditional therapy					
IVIG/CS vs CS	IVIG	Phase 4 Int.	30 actual	ACTRN12612000252819	40412552
Privigen	IVIG	Phase 3 Int.	18 actual	NCT02374736	
Double plasmapheresis vs PLEX	Plasmapheresis	Int.	16 estimated	NCT03436134	
rATG/PLEX vs rATG/PLEX/IVIG ‘INHIBIT’	IVIG	Phase 3 Int.	17 actual	NCT04302805	40458542
Basiliximab vs rATG	IL2	Phase 3 Int.	244 estimated	NCT05385432	
IVIG/rituximab vs rituximab	CD20	Phase 4 Int.	50 actual	NCT04033276	37169835
Bortezomib	Proteasom	Phase 2 Int.	44 actual	NCT01873157	
Carfilzomib and betalacept	Proteasom and CD80/86	Phase 2 Int.	100 estimated	NCT06918990	
Novel data fitting the traditional therapy					
Clazakizumab	IL-6	Phase 2 Int.	20 actual	NCT03444103	33443079
Clazakizumab	IL-6	Phase 1/2 Int.	10 actual	NCT03380377	35497778
Clazakizumab ‘IMAGINE’	IL-6	Phase 3 Int.	194 actual	NCT03744910	10.1681/ASN.2024ktr5vxvn
Tocilizumab ‘INTERCEPT’	IL-6R	Phase 3 Int.	50 estimated	NCT04561986	
Tocilizumab	IL-6R	Observational	10 actual	NCT03859388	
Daratumumab ‘DARTABMR’	CD38	Int. case/control	20 estimated	NCT07081126	
Felzartamab	CD38	Phase 2 Int.	22 actual	NCT05021484	38804514
Felzartamab ‘TRANSCEND’	CD38	Phase 3 Int.	120 estimated	NCT06685757	
Felzartamab ‘TRANSPIRE’	CD38	Phase 2 Int.	81 estimated	NCT07219043	
Isatuximab	CD38	Phase 1/2 Int.	23 actual	NCT04294459	38147137
Mezagitamab	CD38	Phase 1/2 Int.	50 actual	NCT03439280	40552138
GEN3014	CD38	Phase 1/2 Int.	130 actual	NCT04824794	10.1182/blood-2023-179504
Imlifidase	IgG (cleaving)	Phase 2 Obs.	18 actual	NCT04711850	39023092
Imlifidase	IgG (cleaving)	Phase 2 Int.	3 actual	NCT05049850	
Imlifidase	IgG (cleaving)	Phase 2 Int.	30 actual	NCT03897205	
Imlifidase	IgG (cleaving)	Obs.	31 actual	NCT03611621	
Imlifidase ‘CONFIDES’	IgG (cleaving)	Phase 3 Int.	64 actual	NCT04935177	
Berinert^®^	C1	Phase 1/2 Int.	20 actual	NCT01134510	25606785
Berinert^®^	C1	Phase 3 Int.	63 actual	NCT03221842	
Berinert^®^	C1	Phase 1/2 Int.	70 actual	NCT02134314	31843975
Eculizumab	C5	Phase 1/2 Int.	6 actual	NCT01095887	
Eculizumab	C5	Phase 1/2 Int.	11 actual	NCT01895127	
Pegcetacoplan ‘NOBLE’	C3	Phase 2 Int.	13 actual	NCT04572854	39810766
Novel data not fitting the traditional conceptualization					
Mesenchymal stem cell transplantation		Phase 2 Int.	20 estimated	NCT06886646	
Mesenchymal stem cell transplantation		Phase 2 Int.	4 actual	NCT03585855	
FT576	NK cells	Phase 1	31 actual	NCT05182073	10.1182/blood-2022-166994
The new therapies					
Efgartigimod ‘SHAMROCK’	Neonatal FcR	Phase 1/2 Int.	30 estimated	NCT06503731	10.1016/j.ajt.2025.07.1641
Fostamatinib ‘FOSTAMIR’	SYK	Phase 1/2 Int.	8 actual	NCT03991780	
Blinatumomab	CD19/CD3 BiTE	Phase 3 Int.	405 actual	NCT02013167	28249141
Teclistamab	CD19/CD3 BiTE	Phase 2 Int.	194 actual	NCT04557098	35661166
Tegoprubart	CD40L	Phase 2 Int.	132 estimated	NCT06126380	
Tegoprubart	CD40L	Phase 2 Int.	120 estimated	NCT05983770	
Tegoprubart	CD40L	Phase 1/2 Int.	48 estimated	NCT05027906	
Frexalimab	CD40L	Phase 2 Int.	129 actual	NCT04879628	38354138

From left to right: treatment evaluated in the trial and trial name if existing; Immunological target of treatment; study type; participants; trial number; PubMed ID (PMID), if not available: DOI (database: ClinTrials.gov).CD, Cluster of differentiation; CS, corticosteroids; Int., interventional study; Obs., observational study; PLEX, plasma exchange; rATG, anti-thymocyte globulin.

Imlifidase is an IgG-degrading enzyme purified from *Streptococcus pyogenes* that cleaves human IgG into Fab2 and Fc fragments [7]. The destruction of IgG in AMR prevents IgG-mediated phagocytosis, antibody-dependent cellular cytotoxicity and complement-mediated injury [8]. To date it is approved for desensitization therapy pre-transplantation. Evidently, the question arose of whether imlifidase could also be used to treat AMR post-transplantation. Phase 2 trials showed a 97% DSA reduction in the imlifidase group compared with 42% in the plasmapheresis group (primary endpoint), the antibody rebound between 6 and 12 days were similar and a higher rate of graft loss (6/18) occurred in the imlifidase arm vs the plasmapheresis group (1/10). Despite meeting the primary endpoint of maximum DSA reduction compared with plasmapheresis, the trial was unsuccessful in demonstrating a clinical benefit of imlifidase in that indication [8].

Trials on the safety and efficacy of complement inhibitors in AMR yielded ambiguous results. A phase 2 RCT with 20 patients concluded that the C1 inhibitor berinert was safe and reduced post-transplant DSA-levels significantly, yet the small sample size called for further trials [9]. Subsequently, a larger double-blind, randomized-withdrawal, placebo-controlled phase 3 study was terminated early, due to futility of enrolment (NCT03221842). Recent phase 1/2 interventional studies investigating the safety and efficacy of the anti-C5 antibody eculizumab were terminated due to biopsy-proven AMR progression in the treatment arm (NCT01895127) and terminated due to poor enrolment (NCT01095887). C3 inhibitors, interfering earlier in the complement cascade, are being discussed as potential future therapy options. Cp40, a second-generation compstatin analogue that inhibits selectively complement factors C3 and C3b, prevented early AMR and prolonged graft survival in the presence of high levels of DSA in non-human primates [10]. Accordingly, pegcetacoplan, another C3 inhibitor, demonstrated efficacy, safety and tolerability for patients with posttransplant recurrent C3 glomerulopathy [11]. We are awaiting further in-human trials for AMR.

CD38-targeting represents the most promising novel AMR treatment. CD38 is expressed by plasmablasts, plasma cells and natural killer (NK) cells. Depletion of malignant plasma cells with anti-CD38 antibodies has been approved for the treatment of multiple myeloma (MM) [12]. Felzartamab is a monoclonal anti-CD38 antibody, currently under investigation for its use in AMR. In a phase 2 RCT with 22 patients, the antibody proved to be safe, with an adequate side-effect profile and potential clinical benefits, specifically higher rates of AMR resolution, evidenced by lower microvascular inflammation scores, and a more gentle 1-year eGFR slope in the experimental group [13]. The launch of the phase 3 RCT TRANSCEND (NCT06685757) has been announced in early 2025. The trial is currently recruiting patients in Europe and the US to evaluate the efficacy and safety of felzartamab in an estimate of 120 patients. The TRANSPIRE study, registered in late 2025 (NCT07219043), will assess the effects of felzartamab on microvascular inflammation in 81 patients. Isatuximab, another CD38 antibody, was only assessed in an open-label single-arm phase 1/2 trial for efficacy and safety in pre-transplant desensitization therapy which was not sufficiently effective in lowering preformed calculated panel-reactive antibodies [14]. Daratumumab was the first CD38 antibody approved for MM treatment, with multiple case reports existing on its use in AMR [15]. An interventional case–control study (NCT07081126) is currently recruiting 20 AMR patients to test the efficacy of daratumumab compared with IVIG, plasmapheresis and rituximab. A recent small observational study reported that 16 patients diagnosed with AMR showed an overall decrease of DSA, stable eGFR and improved microvascular inflammation scores after receiving daratumumab over the course of 9 months [16]. Interestingly, although the three mentioned antibodies all target CD38, their respective effects on the target cells vary slightly. Felzartamab primarily regulates antibody-dependent cellular cytotoxicity and antibody-dependent phagocytosis. Daratumumab, however, mediates complement dependent cytotoxicity. Isatuximab in comparison, shows the strongest direct pro-apoptotic activity [15]. It could be helpful to consider the heterogeneity of CD38 targeting when establishing a multidrug therapy regimen in kidney transplant recipients, as it may not be reasonable to combine daratumumab with complement inhibitors. Furthermore, a recent study of biopsy bulk transcripts suggested that one main therapeutic effect of felzartamab may be suppression of NK cells also in the graft, rather than plasma cell killing [17]. Additionally, GEN 3014 (NCT04824794) and mezagitamab, both CD38 antibodies, are currently undergoing phase 1/2 trials, however in populations other than AMR patients [18]. GEN 3014 contains a hexamerization-enhancing mutation (E430G) for improved complement activation. Further evidence is still needed to determine whether CD38 targeting will advance AMR treatment, how to best dose CD38 preparations, how treatment response to CD38-directed therapies should be monitored and which drug will ultimately prove to be the most effective.

## NOVEL DATA NOT FITTING THE TRADITIONAL CONCEPTUALIZATION

Interestingly, a novel therapeutic approach in MM involves combining targets of innate and adaptive immunity for targeting malignant plasma cells. FT576 is an NK cell therapy designed to target multiple NK markers. It is made from a genetically engineered stem cell which carries an anti-B cell maturation antigen CAR, a built-in IL-15 fusion protein and a high-affinity, non-cleavable form of CD16. The CD38 gene is completely knocked out to prevent NK cell fratricide and to allow them to work effectively alongside CD38-targeting drugs [19]. FT576 is the first of its kind and currently undergoing a phase 1 trial for dose-finding in MM patients [20]. For the use in kidney transplant recipients, some adjustments may have to be made, since IL-15RF could promote T cell alloreactivity.

Furthermore, we would like to swiftly introduce another possible cellular target for AMR therapy. Bulk transcriptome analyses indicated a substantial contribution of NK cells to microvascular inflammation in AMR. Diebold *et al*. found in an immunohistochemistry study of 86 patients with DSA that the number of NK cells determined by CD34 alongside CD16/T-bet double-staining was significantly higher per mm^2^ area in glomeruli and peritubular capillaries in the 50 biopsies with AMR compared with the 36 without AMR diagnosis per Banff 2019 [21].

## THE NEW THERAPIES

The new AMR treatment may encompass Fc-receptor blockade or spleen-tyrosine-kinase (SYK) inhibition, as well as the introduction of Bispecific T cell Engagers (BiTEs), chimeric HLA antigen receptor (CHAR) T cell therapy and co-stimulatory blockers, of which most have been approved for the use in diseases other than AMR, but could be of potential interest. Efgartigimod alpha is a humanized IgG1-fragment binding the neonatal Fc receptor (FcR), inhibiting IgG–FcR binding and thus recycling, which results in a higher rate of IgG attrition and lower overall IgG levels. It is currently approved for use in patients suffering from myasthenia gravis, and is well tolerated and safe, with the most common side effects being mild headache and mildly reduced monocyte count [22]. The first phase 2 RCT (NCT06503731) investigating the safety and efficacy of efgartigimod in 30 AMR patients is set to be completed in late 2027. Another potential target may be the inhibition of the SYK, which is present in immune cells and putatively important in the process of antibody mediated damage. When activated, SYK phosphorylates downstream scaffolds, leading to higher B cell activation [23]. Fostamatinib inhibits this very kinase and is being evaluated in a phase 2 RCT in AMR (NCT03991780). As of now, roughly 80% of the estimated sample size have been enrolled over the last 6 years.

CHAR T cells represent another intriguing novel idea of AMR treatment. Transduced with a genetically engineered chimeric human leukocyte antigen (HLA) antibody receptor. The goal of CHAR T cell therapy is to exclusively target HLA alloreactive B cells. Three *in vitro* studies showed a very high specificity of CHARs in targeting only those B cell hybridomas producing antibodies with the respective HLA antibody specificity, while sparing those producing other HLA antibodies [24–26]. Additionally, the long-term safety and efficacy of CD19 CAR T cell therapy in refractory systemic lupus erythematosus has been investigated in recent years and yielded promising results, reporting sufficient B cell depletion with limited adverse effects [27]. Furthermore, the use of BCMA CAR T cells for B cell depletion in MM showed superior response rates than the standard treatment and lowered overall disease progression and death [28]. We hypothesize the use of these novel technologies also in AMR, as they may outperform older unspecific B cell depleting treatment ideas, such as CD20 targeted therapy. However, C(H)AR T cell therapy faces several challenges, such as high cost and over-activation by their respective target epitopes, which may trigger cytokine release syndrome [27]. Despite the limitations of C(H)AR-therapy, this approach might facilitate new options for personalized desensitization protocols or AMR treatment. Future *in vivo* AMR trials will tell whether C(H)AR therapy has a relevant future in kidney transplantation.

Ultimately, BiTE is a class of synthetic bispecific monoclonal antibody. Although currently only used in refractory B cell acute lymphoblastic leukaemia, the new antibody blinatumomab is being investigated for potential use in autoimmune disease. Composed of two single-chain antibodies targeting CD19 on B cells and CD3 on T cells fused via a flexible linker, the antibody creates an immunological synapse between CD19+ B cells and T cells, which results in B cell elimination [29]. B cells express CD19 at an earlier developmental stage and retain CD19 positivity longer than CD20, which makes CD19 a more optimal target for B cell depletion [30]. However, blinatumomab therapy may encounter similar hurdles to CHAR therapy, such as cytokine release syndrome and limited efficacy in an immunosuppressed environment, and since all CD19+ B cells are targeted, the risk of infection does increase during therapy [31]. Teclistamab is another antibody employing a similar effector mechanism to blinatumomab. The bi-specific antibody binds CD3 on T cells and BCMA on plasma cells. It is currently approved for the treatment of refractory MM [32]. Although, to our knowledge, there are currently no ongoing or completed trials for the use of blinatumomab or teclistamab in AMR, we see potential for a novel treatment regimen and encourage the realization of phase 2 studies in sensitized kidney transplant recipients.

Lastly, we would like to briefly hypothesize the role of costimulatory signal blockade (CSB), especially CD40–CD40L CSB as a possible new candidate for maintenance immunosuppression with the additional bonus-effect of preventing AMR onset as evidenced in the belatacept trials [33]. Older generations of CD40L inhibitors were unsafe, since they led to platelet activation and subsequent thrombosis. However, the new generation of CD154 antibodies is refined, regarding adverse effects and shows optimistic outcomes in phase 2 studies [34]. Frexalimab is an anti-CD40L monoclonal antibody being evaluated for the treatment of multiple sclerosis, with promising results on safety and efficacy in a phase 2 RCT [35]. Tegoprubart, another CD40L antibody, is currently being investigated for its efficacy and safety in comparison with tacrolimus for maintenance immunosuppression in kidney transplant recipients (NCT06126380). Bestard *et al*. showed in 2011 that a combination of belatacept, sirolimus and rabbit anti-thymocyte globulin sufficiently suppressed T cell allo-response, while maintaining normal numbers of regulatory T cells [36]. This immunomodulatory effect may offer intriguing possibilities for future immunosuppressive therapy regimens and should be considered when investigating novel costimulatory signal blockers.

We are living in exciting times as scientists and clinician researchers are actively creating many new drugs for the treatment of AMR, which has accrued little success until now. The expression of immune and allograft cell antigens is highly dynamic and varies according to both the spatio-temporal stage and the underlying pathogenic mechanisms of alloimmune reactions. In the context of AMR, early stages are predominantly characterized by endothelial activation and complement-related antigen expression, whereas advanced or chronic stages exhibit sustained alterations in antigen presentation associated with ongoing tissue injury and remodelling. Over the next few years, we will likely have targeted solutions for many alloimmune-mediated graft injuries that will prolong transplant and patient survival.

## Data Availability

All of the data used to write this review are publicly available and referenced in the manuscript.
